# Medical News

**Published:** 1853-10

**Authors:** 


### MEDICAL NEWS.

*Proceedings of the College of Physicians, of Philadelphia, in reference to the Death of Dr. Chapman.*

CHAMBER OF THE COLLEGE OF PHYSICIANS,
Stated Meeting, August 3, 1853.

The College of Physicians of Philadelphia, through its Committee, Drs. Bell, Ruschenberger, and West, appointed for the purpose, makes the following record of its sentiments on the occasion of the death of Dr. Nathaniel Chapman, Senior Fellow of the College:

The fulness of years and failing health of Dr. Chapman, and his consequent withdrawal from the active discharge of his professional and social duties, gave, like the twilight that precedes the gloom of night, unmistakable notice of his approaching departure. The melancholy event thus anticipated has occurred; and this College now mourns the loss of its venerated and oldest member—of him, in whom was blended the skilful physician with the kind friend, the instructive writer, and the lucid and emphatic teacher, with the paternal adviser.

The great and distinctive merits of Dr. Chapman, as Professor in that Institution with which his name was so honorably connected during nearly half a century, and in which his ascendency was so conspicuous, will doubtless be delineated in due season. They are fully appreciated by numbers in this College, who, in common with thousands from all parts of the United States, listened to his attractive prelections, and formed their medical opinions under his tuition and guidance.

Farther manifestations of his untiring zeal in fostering medical instruction were afforded by his lecture-laburs, for a period of twenty years, in the Philadelphia Medical Institute, a summer school, of which he was the founder, and, from first to last, President.

As editor and journalist, intent on disseminating medical knowledge, Dr. Chapman has strong claims on the esteem of the entire profession in the United States. The periodical created, and for several years conducted by him, still flourishes, the vehicle for a vast number of highly valuable facts, and for the advocacy of sound ethics.

Long will the name of Chapman be associated in the memory of the members of this College, and of the community in which he lived, with the amenities that give a charm and refinement to social intercourse, and with the radiant yet genial wit which illuminated and quickened into gladness all whom he approached.

How many can bear willing and grateful testimony to his generous heart and freely-given hand, ever responsive to the calls of friendship and of charity, to the timid request of the youthful student for a temporary loan, and the faltering plea of old age for permanent support.

In commemoration of so much worth, and as an incitement to the members of this College and of the profession at large to follow in the path of their distinguished associate,

*Resolved,* That a bust and portrait of Dr. Chapman be procured, and placed in the chamber of the College.

The sincere and respectful condolence of the College is at the same time offered to the widow and family of the deceased under their bereavement. Also, *Resolved,* That a copy of the preceding record be communicated to Mrs. Chapman, by the present Committee.

John Bell,
W. S. W. Ruschenberger,
Francis West.

On motion, the foregoing report was approved, and the resolutions contained therein adopted, and the whole directed to be published in the *American Journal of Medical Sciences* and *Medical Examiner.*

From the minutes.

D. Francis Condie,
*Secretary of the College.*

---

Tribute to the Memory of Drs. Chapman and Horner.—The graduates of the Medical Department of U. P., residing at Mobile, Ala., adopted the following resolutions in reference to the recent deaths of Prof. Chapman and Horner:

Whereas, It hath pleased Divine Providence to remove from the sphere of their usefulness our much respected and honored instructors,

Drs. Nathaniel Chapman and Wm. E. Horner; and, whereas, in our opinion such services as they have rendered the cause of Medical Sciences and our "Alma Mater," deserve some especial and lasting tribute; and, whereas, it was our peculiar privilege to receive instruction from their lips, and to have held up before us their bright examples of zeal and devotion to their profession, and high and honorable conduct in their private life—feelings of respect and esteem for them, and pride for the Science which they have so honored, have prompted us to meet together to give form and expression to the sentiments that fill our hearts in view of the bereavement that their deaths have occasioned. Be it therefore

*Resolved*, That in the death of Professors Nathaniel Chapman and Wm. E. Horner, our time-honored Alma Mater has lost two of its most indefatigable teachers, the city of Philadelphia two of its most respected and esteemed citizens, and the Profession of Medicine two of the most zealous contributors to the facts upon which that science has erected an enduring foundation.

*Resolved*, That as former pupils of these distinguished Professors, we especially know how to appreciate the loss that our profession has sustained—and though we know that the rich treasures of knowledge that they have left us as a legacy to Medical Science will perpetuate their names as long as the truths of that science last—still we, the Alumni of the school, with whose glory their names ~~are~~ so inseparably entwined, would do something more to place their services and their virtues as a shining mark by which to direct the steps of future aspirants for the honors and the fame which reward the Zealous, the Industrious and the Faithful in our Noble Science.

*Resolved*, That in furtherance of the above resolution, we do hereby call upon the Alumni of the Medical Department of the University of Pennsylvania, throughout the world, to contribute the sum of ONE DOLLAR each, on or before the 25th of December next, to defray the expense of erecting a suitable monument to their memories, in the University yard in Philadelphia—that such contribution be sent to the Dean or any member of the present Faculty, to be used by them for that purpose so soon as, in their judgment, a sufficient sum shall have been received.

*Resolved*, That all medical journals and all papers which may approve of this object, be requested to give the action of this meeting publicity, and to further the objects of these resolutions as much as may be in their power.

---

Medical Department of Iowa University.—Dr. McGugin has been transferred to the Chair of Pathology, and E. R. Ford, M. D., of Keokuk, has been appointed to the Chair of Obstetrics, thus vacated by Dr. McG. Edward A. Arnold, M. D., of Baltimore, has been appointed to the Chair of Anatomy, vacated by the transfer of Dr. J. C. Hughes to the Chair of Surgery.

ARMY MEDICAL BOARD.—An order from the War Department constitutes an Army Medical Board for the examination of Assistant Surgeons for promotion, and of candidates for appointment in the medical staff of the army. The Board will convene in the city of New York on the first day of December next, and will probably continue in session three or four weeks. Applications must be addressed to the Secretary of War; must state the age and residence of the applicant, and must be accompanied by respectable testimonials of his possessing the moral and physical qualifications requisite for filling creditably, and for performing ably, the arduous and active duties of an officer of the medical staff.

YALE MEDICAL COLLEGE.—Drs. Henry Bronson and Benjamin Silliman, Jr., have been appointed successors of Professors E. Ives and Benjamin Silliman, in the Medical Department of Yale College; the former in the Department of Materia Medica, and the latter in that of Chemistry.

PROSPECTUS OF THE GEORGIA JOURNAL OF THE MEDICAL SCIENCES.—Under the above title, a new Journal is projected, to be published monthly, at Savannah, Ga., under the editorship of GEORGE F. COOPER, M. D. It has our best wishes for its success.

The PENINSULAR JOURNAL of MEDICINE and the Collateral Sciences, is the title of a new and well conducted Monthly, published at Ann Arbor, Michigan, under the editorial control of E. Andrews, M. D.

Professors COBB and BAXLEY have resigned their respective chairs of Anatomy and Surgery, in the Ohio Medical College, Cincinnati, and are to be succeeded by Drs. G. W. Bayless and Asbury Evans, the former as Anatomist and the latter as Surgeon. Dr. Samuel G. Armor has also been appointed Professor of Physiology and Pathology in the same institution.

Dr. R. J. BRECKENRIDGE has been elected to the chair of Materia Medica, in the Kentucky school of Medicine at Louisville, vice Dr. E. D. Force, resigned.

Prof. A. LITTON.—This gentleman has been appointed Chemist to the Geological Survey of the State of Missouri. He still retains, however, the chair of Chemistry in the St. Louis Medical College, which he has filled with such distinguished ability for eight or ten years past.

BUFFALO MEDICAL JOURNAL.—Dr. S. B. Hunt has become associate editor of this excellent journal, to the pages of which he has heretofore contributed.

Dr. Austin Flint.—This gentleman has resigned the chair of Theory and Practice in the Buffalo Medical College, and Dr. Thomas F. Rochester, of New York, has been appointed his successor. Dr. F. still retains his professorship at Louisville.

Medical Department of the University of Vermont.—This Institution has been organized by the appointment of the following Gentlemen: Dr. W. S. Thayer, of Northfield; Dr. W. Carpenter, of East Randolph; Dr. O. Smith, of Montpelier; Dr. E. S. Carr, of Castleton, Vermont; Dr. H. Nelson, of Plattsburgh, New York; and Dr. L. W. Bliss, of Bradford, Vermont.

American Association for the Advancement of Science.—The proceedings of the scientific convention held at Cleveland on the 28th of July, furnish gratifying evidence of the increased interest felt in science, and of the conviction, among its cultivators, of the necessity of associated efforts. Men of science are evidently realizing, in a highly appropriate manner, the dignity and utility of enrolling themselves as members of this body, which already includes so many who have earned an honorable celebrity. Nearly two hundred new members were elected at the recent meeting.

A full review of the proceedings of the association would be out of place in a journal devoted to strictly medical subjects; we may simply notice that after electing B. Pierce, the Cambridge astronomical professor, to the office of president, and Dr. Baird, of the Smithsonian Institute, and Professor St. John, to the offices of perpetual and annual secretary, the association proceeded to divide itself into sections, and to appoint standing and special committees.

Section A embraces mathematics and physics; Section B is devoted to natural history, geology, chemistry, geography and ethnology. Two-thirds of the papers presented were on subjects connected with mathematics and natural philosophy. Dr. Waldo J. Burnett contributed a paper on blood-corpuscle-holding cells, and their relation to the spleen; and a second paper upon the formation and mode of development of the renal organs in vertebrata. Professor Horsford, of Cambridge, presented an essay on the fatal effects of chloroform.

The officers of these two sections are as follows:

*Standing Committee of Section A.*—Professor Lovering, Chairman;

Professor W. M. Chauvenet, Secretary; Professors E. Loomis, H. L. Eustis, J. Winlock.

*Standing Committee of Section B.*—Professor S. St. John, Chairman; Professor H. L. Smith, Secretary; Drs. J. L. Riddle, W. J. Burnett, Jos. Leidy.

We regret to observe that a resolution was passed prohibiting the sale of the "Transactions" of the association. It appears that the annual assessment of one dollar each from six hundred members suffices to defray the expenses of publication, and it has accordingly been determined that the edition of the Transactions shall be limited to a number of copies sufficient to supply the individual members, and foreign learned societies.

The next meeting of the association will be held in Washington on the last Wednesday in May 1854.—*Virginia Med. and Surg. Jour.*

Obituary.—The profession in Richmond deplores the loss of Dr. William J. Clarke, who expired on the 20th of August, in the twenty-ninth year of his age.

Dr. Clarke possessed excellent capacities, and very considerable attainments in medicine. He was the eldest son of the late Micajah Clarke, who enjoyed for many years a wide and well earned reputation as a practitioner of truly wonderful sagacity. In consequence of the immense success of the father, the career of the son has been observed with more than ordinary interest, and his untimely end has elicited the warmest regrets.

Doubtless, had time been given him, had he reached the maturity of his powers, he would have gained enviable distinction, and have worthily succeeded to the great place in public estimation which his father occupied. Such at least were the hopes which his talents and mental cultivation permitted his friends to entertain, notwithstanding the excessive modesty and reserve which impeded his professional advancement. Therefore their grief is the more poignant, that in his early manhood, his work all unfinished, Death has come and cut him down.—*Virginia Med. and Surg. Jour.*

Obituary.—Dr. John M. F. Trudeau, long a resident and respectable practitioner of St. Louis, died suddenly on the 22nd of August, of disease of the heart.

Obituary.—The following physicians fell victims to the late epidemic at New Orleans:—

Dr. A. R. Nye, aged about 26 years, a native of New York.

Dr. Jacobson, aged 40 years, late of St. Louis, Missouri.

Dr. A. C. Robertson, aged 23 years, a native of Nashville, Tenn.

Dr. Friend, aged — years, a native of Petersburg, Va.

Quinine in Yellow Fever.—Our experience during the present epidemic, with the Sulphate of Quinine, has convinced us that large doses of this salt cannot be relied on in the early stages of the attack.

In the commencement of the epidemic, the advocates of large doses of quinine soon found that this article, when given in sedative doses, failed to accomplish a cure, although the febrile symptoms gradually gave way to its use.

As the epidemic progressed, and its type and characteristic symptoms became better known, few, as far as we can learn, ventured to give large and repeated doses of this salt, except in particular instances. In our previous epidemic of Yellow Fever, the quinine practice succeeded best; but it is generally conceded, as far as we could ascertain, that this season it failed in a majority of cases to sustain its previous high reputation, as a powerful curative agent—hereafter, we shall have more to say on this subject.—*N. O. Med. and Surg. Journ.*, Sept. 1853.

The Epidemic of Yellow Fever at New Orleans.—About the 26th of May last, the first case of yellow fever entered the Charity Hospital, and after death, black vomit was found in the stomach. The first fever cases originated among the shipping along the Levee, in the Fourth District, from which point it extended rapidly through the adjacent portion of the town. A large population of unacclimated persons, living in wooden huts, with floors and timbers soaked in water, and half decayed, were seized with the disease in the most malignant form. For some time previously rain had fallen almost daily, and this added to a hot, burning sun, seemed to give strength to the poison, and lent intensity to the disease. The streets in this vicinity, for the most part, were unpaved, or planked, and the culverts, gutters, etc., were filled with water, saturated with filth and decaying vegetable and animal matter. The crowded state of these huts and low wooden tenements, with their floors steeped in mud and water, is admirably calculated to generate and propagate the germ of a disease which had already been sown in their midst.

The habits of these people, (being chiefly Irish and German laborers,) notoriously negligent and filthy, and utterly indifferent to all those precautionary measures which a limited knowledge of the laws of hygiene should suggest, served only to add fuel to the conflagration which was destined to extend its ravages to every portion of our devoted city. Hence, for some time, the yellow fever confined its work of death within particular localities,—but by and by gaining strength by what it fed upon, it began to travel to other and more distant points,—to extend its arms, so to speak, in every direction, until it grasped the Four Districts within its deadly embrace. For some time the hope was entertained that those who paid proper regard to personal comfort and cleanliness—who dwelt in high, airy, and well-ventilated apartments might escape the disease; but this proved a delusion,—it soon became apparent that, as heretofore, the epidemic fever was no respecter of persons,—the master was stricken down with the servant—the mistress with the maid—the proud and wealthy were brought to a level with the humble and needy. All who had not passed through some one of our epidemic seasons were exposed to attacks from the disease. As has been already mentioned, the fever made its appearance in the latter part of May, at least one month and a half earlier than usual, and from the first case up to the present, it steadily increased almost daily, until the mortality per diem exceeded that produced by any epidemic known in the annals of our sanitary history. In recording the fearful ravages of the present epidemic we must not forget that we have remained exempt from any such visitation since 1847, and during this time an immense population of unacclimated persons, both from Europe and the north-western part of our own country, have been accumulating in our city. The number of unacclimated persons in the city, at the breaking out of the epidemic has been estimated at 30,000 souls; but many of these, it is fair to suppose, have left the city to escape the disease.

The type of the epidemic differs but little from that to which we have been subject in former years; and the belief that persons had died of the disease in six and eight hours from the moment of seizure, can readily be explained by a better knowledge of the antecedent history of the case; for on inquiry it would generally be found that such individuals have had slight fever and other symptoms of the epidemic for two or three days previously to taking their bed and calling in medical aid. This surmise gains additional strength from the fact that the attack, in many instances, has been so insidious and destitute of alarming symptoms, that it is with difficulty such persons could be persuaded—could be prevailed upon to submit to the usual restrictive treatment.

It is not strange, therefore, that such cases, which had been neglected for two or three days, in the early and curable stage of attack, should terminate in fatal black vomit, in a few hours after the physician is summoned to the bedside of his patient. So much for the apparent malignancy of the present epidemic. In making the foregoing explanation, we aim not to deny the existence of an occasional case of extreme severity; so severe indeed as to terminate in death in a few hours, in spite of the best efforts of the most skilful physicians and the most careful nursing.

In some instances the system seems so thoroughly saturated with the poison of the disease, from the very moment of seizure, that no system of medication, as yet suggested, seems able to cope with and stay the fatal tendency of the fever. Every medical man who has had much experience in the disease, must remember occasional instances of this kind.

The disease this season, though essentially the same in many of its most prominent features, exacts, perhaps, on the part of physician and nurse, more care, diligence and precaution, to terminate favorably, than usual in our epidemics. The slightest imprudence, either in diet, exposure, or excitement of any kind, is almost certain to superinduce a relapse—from which state it is usually very difficult to extricate the patient. Hence, the great mortality among those who are not only ignorant of the peculiarities of the disease; but who are also unable, and in some instances unwilling to pay for the requisite medical aid and attendance.

We refer to our table below, furnished by Dr. Simonds, the active Secretary of the Board of Health, for a full account of the deaths and other particulars which have occurred since the epidemic broke out. By this, it will be seen that yellow fever has done terrible execution among our unacclimated population; has produced a mortality unparalleled in the history of our ill-fated city. Even while penning these lines, the fever is sweeping off over *two hundred per diem*—and from present appearances, it is likely to continue its fearful ravages for, perhaps, weeks to come.

Our quondam associate, Dr. Fenner will, in due time, give us a full and detailed history of this epidemic, as he did that of 1847, when the disease shall have run its course and done its work of death.

Below, we give the mortality produced by the epidemic, in the city of New Orleans, from the 28th May up to the 26th August, inclusive, for 1853:

*The Epidemic.*

Total number of death by yellow fever and other diseases, from May 28 till date:

| *Week ending.* | | *Total.* | *Yellow Fever.* | *Other Dis.* | *Not stated.* |
|---|---|---|---|---|---|
| May | 28 | 140—110 | 1—1 | 139—139 | |
| June | 4 | 157— | 1 | 156 | |
| " | 11 | 154 | 4 | 150 | |
| " | 18 | 147 | 7 | 140 | |
| " | 25 | 167—625 | 9—21 | 158—604 | |
| July | 2 | 177 | 25 | 152 | |
| " | 9 | 188 | 59 | 139 | |
| " | 16 | 144 | 204 | 140 | |
| " | 23 | 617 | 435 | 182 | |
| " | 31 | 884—2210 | 704—1427 | 138—741 | 42 |
| August | 1 | 142 | 106 | 25 | 11 |
| " | 2 | 135 | 115 | 14 | 6 |
| " | 3 | 146 | 124 | 17 | 9 |
| " | 4 | 166 | 135 | 15 | 10 |
| " | 5 | 150 | 128 | 9 | 13 |
| " | 6 | 238 | 194 | 30 | 14 |
| " | 7 | 209—1186 | 165—967 | 40—150 | 4—69 |
| " | 8 | 219 | 187 | 23 | 9 |
| " | 9 | 201 | 166 | 21 | 14 |
| " | 10 | 230 | 193 | 33 | 4 |
| " | 11 | 233 | 192 | 13 | 18 |
| " | 12 | 207 | 180 | 25 | 2 |
| " | 13 | 214 | 179 | 22 | 13 |
| " | 14 | 232—1526 | 191—1288 | 26—163 | 16—75 |
| " | 15 | 217 | 187 | 24 | 6 |
| " | 16 | 193 | 163 | 19 | 11 |
| " | 17 | 219 | 191 | 21 | 7 |
| " | 18 | 219 | 188 | 22 | 9 |
| " | 19 | 234 | 203 | 15 | 16 |
| " | 20 | 224 | 184 | 29 | 11 |
| " | 21 | 269—1575 | 230—1346 | 24—154 | 15—75 |
| " | 22 | 283 | 239 | 29 | 15 |
| " | 23 | 258 | 220 | 24 | 14 |
| " | 23 | 222 | 188 | 23 | 11 |
| " | 25 | 218 | 186 | 19 | 13 |
| " | 26 | 193—1070 | 151—884 | 29—124 | 13—66 |
| Total | | 8336 | 5934 | 2075 | 327 |

N. B.—The retnrns from the St. Patrick's Cemetery since the 31st

July, not having been duly made, cannot be relied on, except for two weeks, when the books were resorted to by the Secretary, to enable him to make a weekly report.—*N. O. Med. and Surg. Journ., Sept.*, 1853.

